# The psychological impact of the COVID-19 pandemic on the Sudanese healthcare workers in quarantine centers: a cross-sectional study 2020–2021

**DOI:** 10.1186/s43045-023-00281-w

**Published:** 2023-02-23

**Authors:** Malaz Hassan Dafaalla Idrees, Moez Mohammed Ibrahim Bashir

**Affiliations:** Faculty of Medicine, University of Khartoum, ElQasr Ave, Khartoum, Sudan

**Keywords:** Anxiety, Depression, Healthcare workers, Sudan, COVID-19 pandemic

## Abstract

**Background:**

After its emergence in Wuhan in December 2019, the COVID-19 virus disseminated around the globe creating an incredible panic and outweighing the healthcare system’s capacities. As a result of this hazardous situation, healthcare workers were placed at an unprecedented challenge making them vulnerable to a wide spectrum of mental health issues including anxiety and depression. This was a cross-sectional health facility-based study aiming to explore the psychological impact of the COVID-19 pandemic on Sudanese healthcare workers in COVID-19 treatment centers. Convenience sampling was applied. And two-part self-administered online questionnaire was used: the first part consisted of the demographic data and concerns related to COVID-19, and the second part consisted of the Hospital Anxiety and Depression Scale (HADS) standardized questionnaire. Ethical clearance was obtained from the Ministry of Health.

**Results:**

One-hundred thirty-three healthcare workers of different job titles were enrolled in this study. A total of 54% were females, and 46% were males. Most of the participants had borderline depression (44%) or anxiety scores (47%). Females had significantly a higher mean anxiety score than males. There was no significant difference between participants with and without chronic disease on both anxiety and depression scores.

**Conclusions:**

Healthcare workers at the forefront of the fight against COVID-19 pandemic are facing critical mental health challenges. An immediate intervention from the decision-makers is needed to mitigate this deleterious impact and to strengthen the healthcare system’s capacity in the face of healthcare emergencies in general.

## Background

In December 2019, an outbreak of a respiratory illness emerged in Wuhan City, China. The novel coronavirus disease 2019 (COVID19) was identified to be the cause of the illness [1].

On January 30, the World Health Organization emergency committee announced that COVID-19 is a public health emergency of international concern, later described as a pandemic [2].

Working in all kinds of healthcare facilities from COVID-19 treatment centers, urgent care centers, and intensive care units to ambulances and rehabilitation centers. healthcare workers have contributed to a broad array of services throughout the COVID-19 pandemic [3].

This extraordinary and perpetual catastrophe places a great challenge on healthcare workers predisposing them to develop contemporary psychological conditions [4] in addition to the exacerbation of preexisting ones [5].

Symptoms of fear, anxiety, depression, burnout fatigue [4, 6, 7], posttraumatic stress disorder [8], and sleep disturbances [9] have been reported by healthcare workers.

Although healthcare workers have been experiencing such mental challenges all the time with the emergence of the pandemic, a new set of adjustment strategies and occupational measures have come to place. Handling unfamiliar tasks and caring for patients under the circumstances of a disease that is highly contagious and with no exact treatment intensify the work pressure [10].

The contributing factors varied widely as they included factors inherent to healthcare workers such as gender [11], marital status [12], financial status [13], education level, occupation [14], and whether they suffered a chronic disease [15]. Other factors are extrinsic to HCW, such as work loading and shift changes [16], availability of personal protection equipment (PPE) and/or medical resources, perceived risk of infection, social pressure and stigmatization [17], family support, loss of loved ones, fear of transmitting the disease to loved ones, and living apart from one’s family to avoid infecting them [18].

Anxiety, depression, and burnout have detrimental outcomes on healthcare workers combating this crisis affecting their critical thinking abilities and problem-solving skills which might disrupt their health conditions, decrease their quality of life, and impact their work performance [19], thus reducing their capacity to effectively address the healthcare emergency if proper interventions to enhance resilience are not applied [20].

There is a problem of poor documentation combined with the scarcity of information addressing the psychological impact of COVID-19 pandemic on healthcare workers in isolation centers in Sudan.

This study aims to explore the psychological problems in terms of anxiety and depression among healthcare workers in COVID-19 treatment centers in Sudan 2020–2021 specifically the prevalence of anxiety and depression.

Policymakers given their role in responding to the situation need evidence in order to apply the necessary future interventions which are needed to combat or mitigate the negative psychological consequences and strengthen the response capacity of the healthcare system not only with regard to COVID-19 pandemic but healthcare emergencies in general.

The subject of this research is going to provide information that will act as a solid baseline upon which the proper solutions can be conducted.

## Methods

### Study design

This is a descriptive cross-sectional health facility-based study aiming to investigate the psychological impact of COVID-19 pandemic on healthcare workers in isolation centers in Sudan. The study was carried out between December 2020 and January 2021.

### Study setting

This study was conducted in Khartoum state in two isolation centers, Ibrahim Malik (a primary isolation center) and Jabra (a secondary isolation center).

- Ibrahim Malik Hospital: Located in the middle of Khartoum province and is one of the most ancient hospitals in the city which has recently developed a primary pandemic center with a relatively small staff of 21 healthcare workers where patients are admitted for initial stabilization and then referred to the secondary isolation centers which have a better capacity
- Jabra Hospital: It is an emergency and casualty hospital that has been converted into an isolation center in an effort of combating COVID-19 outbreak.

The above two centers were picked owing to their good background and the important role they played during the pandemic as these are the most popular centers with a high turnout that people commonly go to due to their relatively good capacity and preparedness; also, at the time this study has been conducted, Sudan had only five governmental quarantine, some of which were later dissolved into regular hospitals, and only two of them granted permission to conduct the research.

### Study population

This study was conducted among healthcare workers at two Sudanese isolation centers: Ibrahim Malik Primary Isolation Center and Jabra Secondary Isolation Center, with a total of 178 healthcare workers in both sites (a total of 157 healthcare workers in Jabra Secondary Isolation Center and 21 healthcare workers in Ibrahim Malik Primary Isolation Center).

#### Inclusion criteria

- All healthcare professionals working in the two centers in all different departments including medical staff, nursing staff, laboratories, infection control, radiology, and biomedical who agreed to participate in the study.
- Both males and females were enrolled.
- Aging 18 and above

#### Exclusion criteria

- Nonmedical personnel
- Healthcare professionals who have been previously diagnosed with other psychiatric illnesses were excluded in order to rule out the effect of factors other than COVID-19 pandemic.

### Sample size and sampling

Regarding the population in Jabra Secondary Isolation Center, convenience sampling was used, and the sample size was calculated using the following formula:

- *n* = N/1+(Ne2)
- *n* = 157/1+(157*0.05^2) = 112

The estimated acceptable margin of error was 0.05 based on the mentioned formula, the sample size was estimated to be a minimum of 112 participants, and therefore, 112 healthcare workers were selected from Jabra Secondary Isolation Center.

Because the total population at Ibrahim Malik Primary Isolation Center was already relatively small (21 healthcare workers), total coverage was used, and thus, all 21 healthcare workers were chosen for the study.

The total sample in this study (*n*) was therefore 133 healthcare workers.

### Instruments of measurement

An online self-administered questionnaire created through Google Forms was sent to the participants on WhatsApp or Telegram as the data collection was conducted during the period of lockdown.

The questionnaire embodied two parts: the first part entailed socio-demographic data and questions concerning the COVID-19 pandemic. The second part was comprised of the Hospital Anxiety and Depression Scale (HADS).

#### Demographic data

The demographic data included age, sex, occupation, and working place.

#### Questions pertaining to the participants’ concerns with regard to COVID-19 pandemic

These questions involved the perceived risk of hospital-acquired infection, the presence or absence of chronic disease, worry about losing a beloved one to the virus, worry related to the shortage of personal protective equipment, and a self-evaluation question to assess the workload during the pandemic.

#### The hospital anxiety and depression scale

A self-administered scale designed to screen for both anxiety and depression was used. It is a 14-item scale (7 for each anxiety and depression) with a score ranging from 0 to 21.

A score of less than 7 is considered normal, 8–10 mild, 11–14 moderate, and 15–21 severe.

This scale has been used owing to the fact that it has proven to be psychometric valid and reliable which makes it a useful and easily applicable tool to screen for anxiety and depression in clinical practice [21–25].

### Data collection management and analysis

Questionnaires were refined and managed carefully; completeness was checked before data entry. Data generated on the questionnaire were numbered and entered after clearing and refined for analysis using Statistical Package for Social Science (SPSS) version no. 23 software for statistical analysis and presented as frequencies and percentages. An independent sample *t*-test was used to compare anxiety and depression between different groups with *p*-value less than 0.05 considered significant.

## Results

**Fig. 1:**
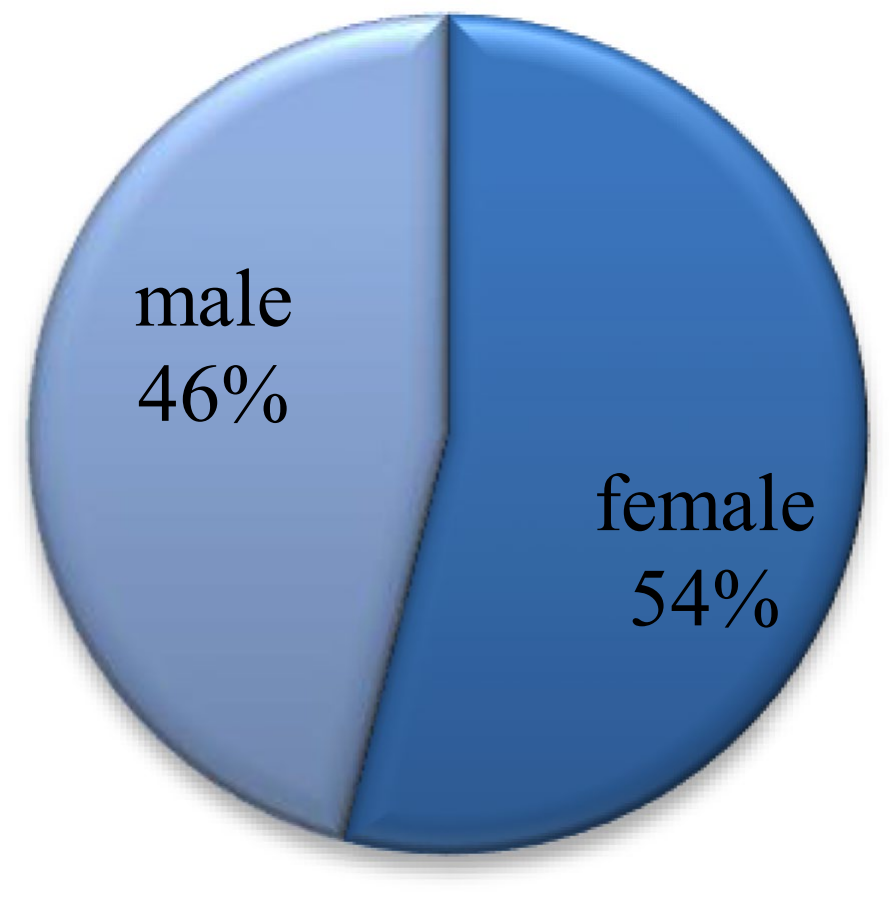
Gender distribution among participants. Shows the percentage of males and females who participated in the study. The males were 46%, and the females were 54%

One-hundred thirty-three healthcare workers have been enrolled in this study, 54% of whom were females (Fig. 1).

The mean age of the participants was found to be 27 years (*SD* = 3.5) (Table 1).

**Table 1:** Sociodemographic characteristics and working conditions. Shows the gender, the workplace, the occupation, the presence or absence of chronic disease, and the workload during the pandemic

		Frequencies (%)
Gender	**Female**	**72 (54%)**
	**Male**	**61 (46%)**
The workplace	**Jabra Isolation Center**	**112 (84%)**
	**Ibrahim Malik Isolation Center**	**21 (15%)**
Occupation	**Medical staff**	**61 (46%)**
	**Nursing staff**	**54 (41%)**
	**Laboratories**	**4 (3%)**
	**Biomedical engineering**	**1 (0.8%)**
	**Radiology**	**2 (1.5%)**
	**Infection control**	**8 (6%)**
	**Pharmacy**	**3 (2%)**
Chronic diseases	**Present**	**15 (11%)**
	**Absent**	**118 (89%)**
Workload during pandemic	**Significantly lower than before**	**2 (1.5%)**
	**Lower than before**	**10 (7.5%)**
	**The same as before**	**12 (9%)**
	**Higher than before**	**53 (40%)**
	**Significantly higher than before**	**56 (42%)**

**Fig. 2:**
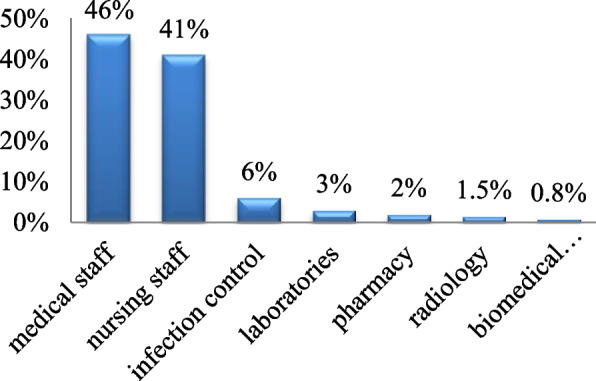
Occupation. Shows different professions pursued by the participants

The majority of the participants belonged to the medical staff (46%) followed by the nursing staff (41%), infection control (6%), laboratories (3%), pharmacy (2%), radiology (1.5%), and biomedical engineering (0.8%) (Fig. 2).

**Fig. 3:**
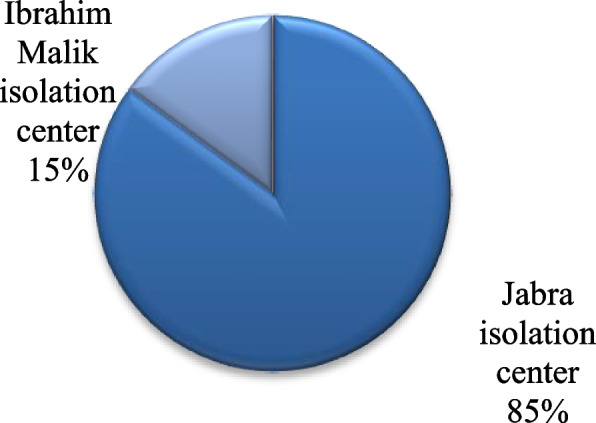
Workplace. Shows the workplace of the participants. A total of 15% of them work in the Ibrahim Malik Isolation Center

The majority of the participants work at Jabra Isolation Center (85%), whereas 15% of them work at Ibrahim Malik Isolation Center (Fig. 3).

**Fig. 4:**
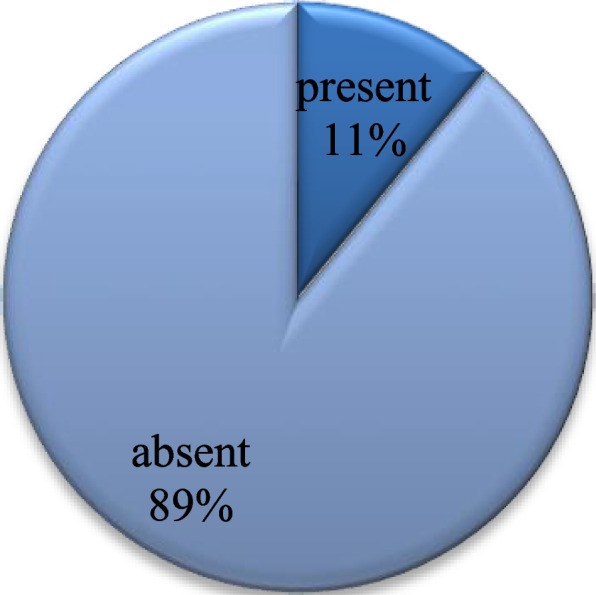
Chronic disease. Shows the percentage of participants with and without chronic disease. Being absent in 89% and present in 11%

**Fig. 5:**
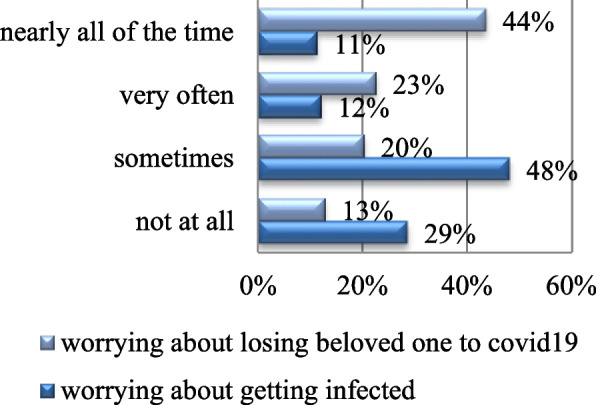
Concerns. Shows concerns pertaining to the COVID-19 pandemic. A total of 44% reported that they worry about losing a beloved one to COVID-19 nearly all the time, whereas 48% worry about getting infected sometime

**Fig. 6:**
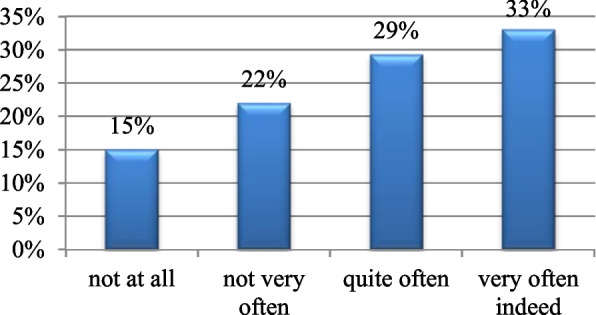
Personal protective equipment. Shows concerns related to the shortage of personal protective equipment. A total of 33% reported that they worry very often indeed, whereas 15% do not worry at all

A total of 89% of the participants denied suffering from any chronic disease (Fig. 4). A total of 42% of them had a significantly higher workload than before the pandemic (Table 1). A total of 44% of the participants have worries pertaining to losing a beloved one to COVID-19 all the time, 48% worry about getting infected sometimes (Fig. 5), and 33% showed worries related to the shortage of personal protective equipment (Fig. 6).

The mean anxiety and depression scores were 8.8 for both.

**Fig. 7:**
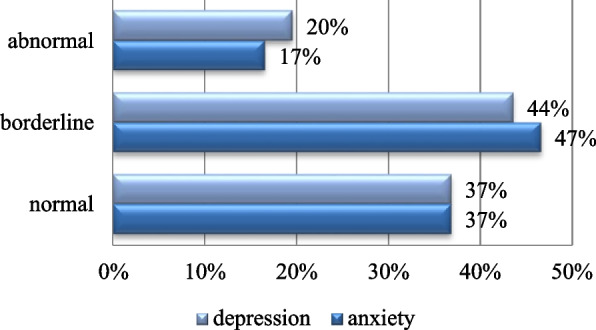
Anxiety and depression scores. Shows that 44% of the participants had borderline depression score. A total of 47% had borderline anxiety score

As per the HADS score, most of the participants had borderline depression (44%) and anxiety scores (47%). A total of 17% had abnormal anxiety scores, 20% had abnormal depression scores, and 10% had abnormal anxiety and depression scores at the same time (Fig. 7).

Females had significantly high mean anxiety scores than males (95% *CI* 0.4–3.1) (*p*-value 0.01) (Table 2).

**Table 2:** Comparison of gender and chronic disease with anxiety score. Shows that females had an anxiety score of 9.7 in comparison with males’ 7.9. No significant difference between males and females in anxiety score

	Mean anxiety score (SD)	*p*-value	95% confidence interval	
			Lower	Upper
Gender				
Female	**9.7 (4.2)**	0.011*	**0.4**	**3.1**
Male	**7.9 (3.6)**			
Chronic disease				
Present	**9.3 (3.9)**	**0.65**	**-1.7**	**2.7**
Absent	**8.8 (4)**			

There was no significant difference between participants with and without chronic disease on both anxiety and depression scores (Tables 2 and 3).

**Table 3:** Comparison of gender and chronic disease with depression score. Shows that there is no significant difference in depression in patients with and without chronic disease

	Depression score (SD)	*p*-value	95% confidence interval	
			Lower	Upper
Gender				
Female	**9.4 (3.7)**	**0.06**	**−0.03**	**2.4**
Male	**8.1 (3.3)**			
Chronic disease				
Present	**9.7 (3.9)**	**0.3**	**−0.9**	**2.9**
Absent	**8.7 (3.6)**			

## Discussion

This study is one of the pioneer studies that explore the psychological impact of the COVID-19 pandemic on the healthcare workers in Sudan especially that it sheds light specifically on the healthcare professionals working within an exceptional environment such as that of the quarantine centers.

According to the Hospital Anxiety and Depression Scale (HADS), 44% of the respondents showed borderline depression scores, and 47% had borderline anxiety scores. The mean anxiety and depression scores were 8.8.

Females had significantly a higher mean anxiety score than males, with no major difference between participants with chronic diseases and without diseases on both anxiety and depression scores.

These findings are found to be consistent with the outcomes of a study conducted to evaluate the psychological status of healthcare professionals working in Egypt and Saudi Arabia in terms of anxiety, depression, stress, and sleep quality. Four-hundred twenty-six healthcare workers have been included; of them, 69% had depression, and 58.9% had anxiety [26].

A noteworthy finding in this study is that 29% of the participants denied any fear of contracting the virus, 48% of them revealed that they fear getting infected “sometimes,” and only 11% fear contracting the virus “nearly all the time.” This might be the result of developing coping mechanisms after almost more than a year of working in a zone where there is a great risk of infection. Another possible explanation is that the majority of the participants are of a young age (the mean age is 27), and it has been reported that young age groups suffer milder symptoms of the disease. In a prospective study conducted on patients with flu-like symptoms referred to the imaging department of a tertiary hospital in Iran between March 3, 2020, and April 8, 2020, age seemed to be the most important risk factor for COVID-19; the study demonstrated that comorbidities such as cancer and underlying heart diseases are associated with a higher COVID-19 mortality rate [27].

Increased work pressure during the pandemic has also been identified as a risk factor; in this study, 42% of the respondents deem the workload to be “significantly higher than before,” and 40% of them find it “higher than before.” These outcomes are reinforced by a study that has been conducted to evaluate the impact of the COVID-19 pandemic on the workload and mental health of Iranian medical staff, in which it has been confirmed that health workers who encountered COVID-19 patients were subjected to more task load compared to those who had no contact with COVID-19 patients at the workplace [28].

There is a significant amount of worry related to the unavailability of personal protective equipment; in the present study, it has been shown that 33% of the respondents had a highly significant worry regarding the shortage of personal protective equipment. Furthermore, this finding is strengthened by the outcome of an observational cross-sectional, questionnaire-based survey carried out in Pakistan in which 70.2% of the participants were afraid of not having proper protective equipment [29].

### Limitations

The study had several limitations related to the restricted access to the participants due to the nature of their work and the relative risk of infection.

As the study was conducted during the period of lockdown, an online survey in which only one scale to screen for the symptoms of anxiety and depression has been used as asking healthcare workers in the arduous environment of the COVID-19 treatment centers too many questions was not feasible. In addition to this, the outcomes were self-reported which largely depended on the participants’ memory and concentration making them prone to recall bias.

This study was meant to include around 5 COVID-19 treatment centers but has been limited to only two centers due to feasibility issues, uncooperative administrative boards of some centers, and the dissolution of others.

## Conclusions

The study concluded that COVID-19 pandemic has placed a great challenge on the mental health of healthcare workers. A considerable percentage of healthcare workers suffer from borderline anxiety and depression. Females had significantly a higher mean anxiety score than males. There was no major difference between participants with chronic diseases and without diseases on both anxiety and depression scores. Findings showed that there were concerns related to the shortage of PPE and worry about loved ones. An appreciable percentage of healthcare workers denied any fear related to contracting the infection.

It has been well established in the literature that anxiety, depression, and burnout have detrimental effects on healthcare workers; our research findings directly suggest that the Sudanese healthcare professionals are more likely to have reduced work capacity, work performance, and lower quality of life as a result of the COVID pandemic’s effect on their mental health.

## Data Availability

The datasets used and analyzed during the current study are available from the corresponding author upon reasonable request.
